# The Effect of Distraction Osteogenesis on Peripheral Nerve Regeneration in Rats: A Preliminary Study *In Vivo*

**DOI:** 10.1155/2023/8818561

**Published:** 2023-06-14

**Authors:** Kai Liu, Yuanxin Chen, Feiyu Cai, Xin Wang, Chenchen Fan, Peng Ren, Aihemaitijiang Yusufu, Yanshi Liu

**Affiliations:** ^1^Department of Trauma and Microreconstructive Surgery, The First Affiliated Hospital of Xinjiang Medical University, Urumqi, Xinjiang 830054, China; ^2^Uygur Medical College, Xinjiang Medical University, Urumqi, Xinjiang 830011, China; ^3^Department of Orthopaedics, The Affiliated Hospital of Southwest Medical University, Luzhou, Sichuan 646000, China

## Abstract

Distraction osteogenesis (DO) is a widely employed method for the treatment of limb discrepancies and deformity correction. This study aimed at observing the histomorphological and ultrastructural changes of peripheral nerves around the distraction area during DO and investigating the self-repair mechanism of peripheral nerves in a rat DO model. Sixty rats underwent right femoral DO surgery and were randomly separated into six groups: Control (latency, no distraction, *n* = 10), Group 0-week (after distraction, *n* = 10), Group 2-week (*n* = 10), Group 4-week (*n* = 10), Group 6-week (*n* = 10), and Group 8-week (*n* = 10) at consolidation phase. The right femur of rats in Group 0-week, Group 2-week, Group 4-week, Group 6-week, and Group 8-week was subjected to continuous osteogenesis distraction at a rate of 0.5 mm/day for 10 days. Motor nerve conduction velocity (MNCV) of the sciatic nerve, sciatic function index (SFI), histological analyses, and transmission electron microscopy were conducted to evaluate nerve function. The MNCV and SFI of Group 0-week, Group 2-week, Group 4-week, and Group 6-week were significantly lower than the Control (*P* < 0.05). No statistical differences were found between the Control and Group 8-week in terms of MNCV and SFI (*P* > 0.05). Injuries to nerve fibres and nodes of Ranvier were observed in the Group 0-week, whereas the nerve fibres returned to the normal arrangement in the Group 8-week and oedema of myelin disappeared, with the continuity of axons and lamellar structure of myelin being restored. Femoral DO in rats with a rate of 0.5 mm/day may cause sciatic neurapraxia, which can be self-repaired after 8 weeks of consolidation. The paraneurium around the sciatic nerve enables it to glide during the distraction phase to reduce the occurrence of injurious changes.

## 1. Introduction

Distraction osteogenesis (DO) has been widely used in the treatment of limb discrepancies and deformity correction [1, 2]. Intramembranous bone formation is induced by gradual mechanical distraction via an external fixator during DO, and this osteogenic process is usually regulated by the differentiation of bone mesenchymal stem cells, hypoxia-inducible factors, and dynamic mechanical microenvironment [3, 4]. Previous studies have reported that a moderate mechanical microenvironment has benefits in promoting bone formation by activating the HIF pathway and subsequently augmenting the osteogenic-angiogenic coupling [5]. However, few studies have focused on the phenomenon of peripheral nerve apraxia and self-repair during DO, which may involve the process of bone regeneration and remodelling.

Previous studies have reported the subclinical damage of peripheral nerves around the distraction area caused by DO [6–8]. Theoretically, nerves and soft tissues around the distracted area are inevitably affected during the distraction phase. However, there is a layer of undulating bundles of collagen fibres and elastic fibres in the interfascicular epineurium which makes the peripheral nerve naturally elastic and allows the fascicles to glide against one another [9, 10]. Although the collagen fibres of peripheral nerves can exhibit self-repair over a low-deformation range, they will be irreversibly damaged if the velocity exceeds the elastic limitation (>1 mm/day) [11, 12]. For instance, studies on mandibular DO have reported that a higher stress load may severely affect the synthesis and proliferation of neural cells, which may lead to neuroma and neurodegeneration [6, 13].

Mitogen-activated protein kinase (MAPK) cascade is composed of MAPK kinase kinase (MAP3K), MAPK kinase (MAP2K/MEK/MKK), and MAPK in response to various extracellular stimuli. P38 MAPK is one of the major members of the MAPK family and is involved in the regulation of cellular proliferation, differentiation, and death of all eukaryotic cells from yeast to humans. The MAP3K-MAP2K (MKK)-MAPK pathway is the most common pathway that can be activated, representing a tertiary enzyme-linked reaction. Studies have confirmed that p38 MAPK is involved in the regulation of cellular differentiation at the MAPK cascade, which is closely related to the development of neural and skeletal systems in humans [14, 15]. Although p38 MAPK is often silent, ischemic, hypoxic, and inflammatory factors can activate it and lead to its phosphorylation (p-p38) and subsequent entry into the cellular membrane or nucleus. Beyond this, some studies have found that p38 MAPK can be further involved in the process of cellular growth in the human body and is involved in the response to inflammation and cellular stress. Moreover, it can be involved in the repair and regeneration of cells and the related processes of its regulation [16, 17], such as the MAPK/NF-*κ*B [18] and MAPK/ERK signalling pathways [19]. P38 MAPK not only transmits and transforms a variety of bio signals in the process of cellular regulation, but also interacts with different signalling pathways in the cell, ultimately converging various signal transmission information and becoming the final intersection point [20], making it an important target in the field of neural and bone tissue regeneration [5, 21].

Therefore, the purpose of this study was to observe the histomorphological and ultrastructural changes of peripheral nerves around the distraction area during DO and to investigate the self-repair mechanism of peripheral nerves in a rat DO model.

## 2. Materials and Methods

### 2.1. Animals

Sixty 12-week-old specific-pathogen-free (SPF) male Sprague–Dawley (SD) rats weighing 300 to 400 grams were utilized in this study, which was provided by the Laboratory Animal Center of Xinjiang Medical University (certificate number: SYXK (Xin) 2018-0003). All experimental rats were fed for two weeks in an appropriate environment (22 ± 2°C temperature; 40–60% relative humidity) before undergoing DO surgery, with access to healthy food and water. The ethical standards of animal medicine in China and the ethical standards of the Laboratory Animal Center of Xinjiang Medical University (approval number: IACUC-20200318-82) were strictly followed.

### 2.2. Surgical Technique

After successful general anesthesia with 2% pentobarbital sodium (3 mg/100 g, Sigma, US), the animals were placed on the experimental operation table in the left lateral decubitus position. Skin preparation was performed in the operation area. According to a previous study [3], a longitudinal incision was made on the lateral middle segment of the right thigh. The skin and subcutaneous tissue were carefully incised layer by layer and the space between the vastus lateralis and rectus femoris muscles was opened. The middle line of the right femur was then exposed, which was chosen as the osteotomy level. A preoperatively designed microexternal fixator was placed on the lateral side of the femur and two Kirschner wires (1.2 × 250 mm) were inserted into the proximal and distal parts of the femur, respectively. Osteotomy was then performed using a micro-oscillating saw, and the microexternal fixator was assembled and adjusted for distraction and compression. Finally, the surgical area was flushed with 0.9% saline and the incision was sutured.

### 2.3. Postoperative Care

After surgery, each rat was fed in a single clean cage. Intramuscular injection of benzylpenicillin (200,000 IU/kg) was performed daily for three days to prevent infection. The skin incision was disinfected with 75% alcohol once a day for five days postoperatively. The latency phase lasted for five days, and the distraction phase was started manually with a rate of 0.5 mm/day for ten days. The consolidation phase started upon the completion of the distraction phase. Control (*n* = 10) was defined as no distraction after DO surgery. Group 0-week (*n* = 10), Group 2-week (*n* = 10), Group 4-week (*n* = 10), Group 6-week (*n* = 10), and Group 8-week (*n* = 10) were defined as different time points during the consolidation phase. After a short period of isoflurane anesthesia, all experimental rats underwent weekly anterior-posterior X-ray imaging of the distraction area until they were sacrificed (*n* = 10 per group).

### 2.4. Electrophysiology

Electrophysiology analysis (Alpine/Biomed-Keypoint-4, Dantec Dynamics A/S, Denmark) of the sciatic nerve was, respectively, performed for all groups under general anesthesia (hypnorm and diazepam, *n* = 10 per group). The stimulating electrode was placed at the proximal side of the femur (approximately 2 cm from the distraction area), and an inductive electrode was placed on the innervated muscle. The electromyography rectangular pulse was designed with the following pulse parameters: 0.1 ms, 15.8 mA, 1.5 Hz, and 6 consecutive pulses. The time taken for the conduction of the stimulus was measured and the motor nerve conduction velocity (MNCV) was calculated as the distance between the two stimulating points divided by the difference in the latency of the two points' stimulus.

### 2.5. Sciatic Function Index

A self-made rat walking box (50 × 15 cm) was used to record the four to five footprints of rats during crawling with ink dipped in dye on both feet, and clear footprints on the experimental (E) and normal (N) sides were selected to measure three variables: footprint length (PL), defined as the longest distance of the footprint; toe width (TW), the distance from the first toe to the fifth toe; and intermediate toe distance (IT), the distance from the second toe to the fourth toe. The sciatic function index (SFI, *n* = 10 per group) was calculated by taking the above three variables into account with Bain's formula, SFI = −38.3 (EPL − NPL)/NPL + 109.5 (ETW − NTW)/NTW + 13.3 (EIT − NIT)/NIT − 8.8. A normal functioning limb was considered to have values of SFI closer to 0.

### 2.6. Histomorphometric Analyses of Nervous Tissue

Static histomorphological observation of the sciatic nerve was conducted using Hematoxylin and Eosin staining (H&E, *n* = 3 per group). Specifically, specimens were randomly selected from each group and then fixed in 10% neutral buffered formalin for twenty-four hours. After paraffin embedding and molding, all selected specimens were sliced into ultrathin sections with an ultramicrotome (Leica, Germany) with a thickness of 5 *μ*m, which were stained with H&E stain. The slides were then washed three times with distilled water and dehydrated using a series of increasing concentrations of ethanol. Finally, the slides were air-dried at room temperature and mounted with neutral gum for later observation under an optical inverted microscope (DP26, OLYMPUS Corporation, Japan).

Ultrastructural changes of the sciatic nerve around the distraction area (*n* = 2 per group) were assessed using transmission electron microscopy (Hitachi, Japan). Three samples from each group were fixed in a 2.5% glutaraldehyde solution (Millipore Sigma, US) for transmission electron microscopy analysis. All selected nerve specimens were fixed in 1% osmium tetroxide for one hour and then diluted to 2% for refixation. The selected specimens were rinsed thrice with PBS and dehydrated in a series of absolute ethanol, which were then placed in pure acetone and Epon 812 epoxy solution for thirty minutes and embedded in Epon 812 epoxy at 60°C for forty-eight hours. Ultrathin (70 nm) sections were cut and stained with lead and uranium.

The remaining specimens (*n* = 5 per group) were fixed in 10% buffered formalin. After deparaffinization in xylene and rehydration in a graded series of alcohol, immunohistochemistry staining was performed using a primary antibody against p38MAPK (1 : 100, ab216842, Abcam, UK) at 4°C overnight. The sections were then incubated with secondary rabbit anti-rat IgG (DS-0003, 1 : 100, Zhongshan Golden Bridge Biotechnology, China) for 30 minutes at 37°C. Diaminobenzidine and hematoxylin were used to stain these sections, which were then sealed with neutral resins. Images were captured under an optical inverted microscope (magnification, ×200; DP26, OLYMPUS Corporation, Japan), and the integrated optical density of p38MAPK expression in three randomly selected fields of distraction area was measured using Image Pro-Plus 6.0 software (Media Cybernetics Inc., US). The results were presented as the area percentage of positive staining.

### 2.7. Statistical Analysis

The data were expressed as mean ± standard deviation (SD). The Kolmogorov–Smirnov test was employed to assess the normality of the data. Student's *t*-test was used to compare differences between the two groups, while one-way ANOVA was used for multiple comparisons. In cases where the test of homogeneity of variances yielded a *P* value greater than 0.05, Tukey's multiple comparison post hoc test was applied. Otherwise, Dunnett's T3 post hoc test was used. Statistical significance was considered when the *P* value was less than 0.05 between groups. All analyses were performed by the SPSS 20.0 software package (IBM, US). Graphs were plotted by GraphPad Prism 8.0 software (GraphPad, US).

## 3. Results

### 3.1. Electrophysiology and SFI Analysis

**Figure 1:**
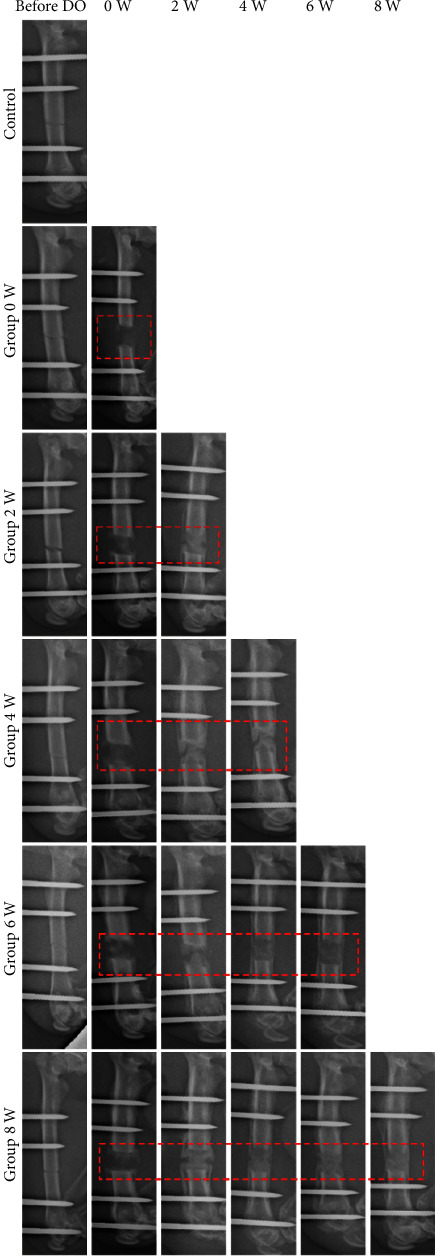
Radiographs of rat femur after DO surgery using external fixation (*n* = 10 per group).

**Figure 2:**
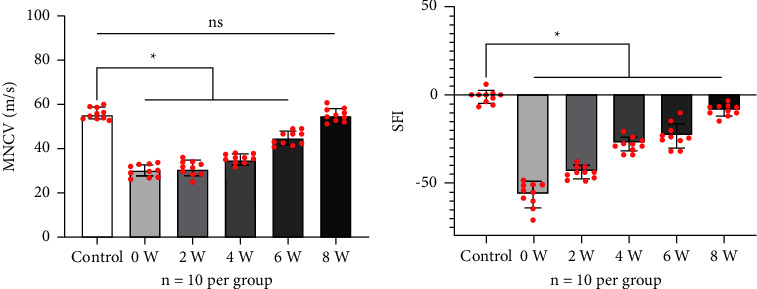
Results of electrophysiology and SFI in different groups (*n* = 10 per group). (a) The motor nerve conduction velocity (MNCV) of the Group 0-week was significantly lower than the other groups (*P* < 0.05), and MNCV increased with increasing duration of consolidation. (b) The sciatic function index (SFI) of the Group 0-week was significantly lower than the other groups (*P* < 0.05), and the absolute value of SFI decreased with the increasing duration of consolidation.

All animals recovered from DO surgery and survived until the termination of the experiment. No walking disturbances were observed in all animals during latency. Radiographs of the right femur indicated signs of the distraction area (Figure 1), which dynamically showed the bone regeneration of the distraction area. MNCV significantly decreased within the Group 0-week (29.24 ± 2.75 m/s), Group 2-week (30.29 ± 5.13 m/s), Group 4-week (31.67 ± 1.85 m/s), and Group 6-week (45.09 ± 3.22 m/s) after the distraction phase (Figure 2), compared to the Control (56.18 ± 2.51 m/s, *P* < 0.05). MNCV of the above groups gradually increased over time and returned to normal at 8-week consolidation (55.38 ± 3.16 m/s). There was no significant difference in MNCV between Group 8-week and the Control (*P* > 0.05). The decrease of MNCV in Group 0-week, Group 2-week, Group 4-week, and Group 6-week, respectively, accounted for 98.6%, 98.5%, 53.9%, 52%, and 52% of the mean MNCV of Control.

SFI was significantly decreased within Group 0-week (−56.9 ± 7.06), Group 2-week (−44.3 ± 3.59), Group 4-week (−28.4 ± 4.08), Group 6-week (−23.3 ± 6.88), and Group 8-week (−8.6 ± 3.37), compared to Control (−4.2 ± 3.76, *P* < 0.05). Similarly, SFI increased throughout the consolidation period and returned to normal at 8 weeks of consolidation (Figure 2). The decreased values of SFI in Group 0-week, Group 2-week, Group 4-week, and Group 6-week, respectively, accounted for 56.9%, 44.3%, 28.4%, 23.3%, and 8.6% of the Control.

### 3.2. Histomorphometric Analysis

**Figure 3:**
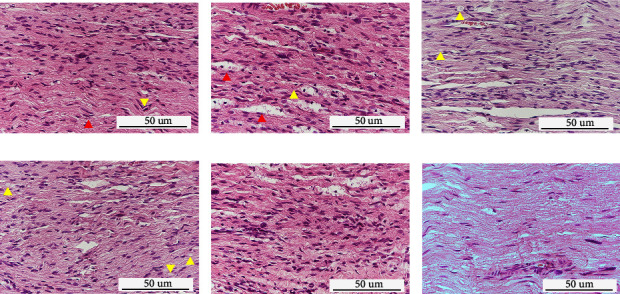
Histological results of the sciatic nerves in different groups (HE staining, *n* = 3 per group). (a) Wavy running nerve fibres were observed in the control, as characterized by the obvious nodes of Ranvier (red arrow) and scattered distribution in Schwann's cell nuclei (yellow arrow). (b) Disorganized nerve fibres were observed in Group 0-week with the widened distance between some nerve bundles (red arrows) and medullary spheroid changes appearing to be a lipid droplet-like structure (yellow arrows). (c) Mild reparative changes were seen in Group 2-week with still-persisting lipid droplet-like structures (yellow arrows). (d) Self-repaired nerve fibres were observed in Group 4-week with myeloid-like changes still present (yellow arrows). (e, f) At Group -week and Group 8-week, the nerve fibres had recovered to the normal arrangement, with the clear observation of the nerve axon, the node of Ranvier, and internode structures.

In the HE staining view of the Control (Figure 3(a)), the sciatic nerve fibres showed a wavy course in the nerve fascicle, and the fascicle was smooth and closely arranged. The axons were also arranged in parallel with good continuity, and nodes of Ranvier were observed prominently. A few darkly stained blue and flattened nuclei of Schwann cells were seen scattered. However, the sciatic nerve fibres of the distraction area were disorganized and nodes of Ranvier were widened when the distraction phase finished (Figure 3(b)). A few lipophilic structures produced by Schwann cell disintegration were observed between the nerve fibres, and the myelin sheath was swollen. Even the nerve fibre expansive demyelination was observed and myeloid-like changes formed by demyelination were noticed as well. Over the consolidation phase, mild reparative changes of sciatic nerve fibres in the distraction area were observed (Figures 3(c)–3(e)). After 8 weeks of consolidation, the arrangement of injured sciatic nerve fibres recovered to a natural and neat wavy shape without disturbance (Figure 3(f)). The structure of nerve axons and nodes of Ranvier was clear and the myelin swelling disappeared, which was approximately similar to the Control.

**Figure 4:**
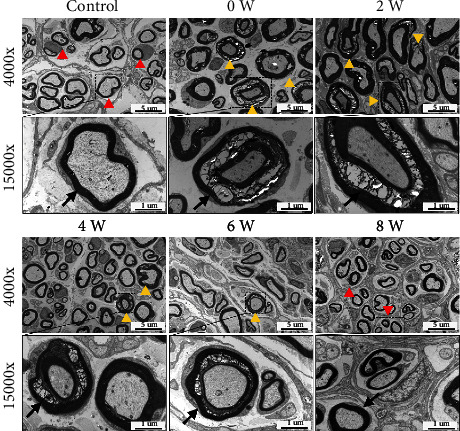
Transmission electron microscopy results of the sciatic nerves in different groups (*n* = 2 per group). In the Control, closely arranged lamellar myelin structures (red arrows) were observed with the axonal membrane being smooth and intact containing microtubule microfilaments. At Group 0-week, the myelin lamellae of myelinated nerve fibres were loosely sheeted apart (yellow arrows), with oedema of Schwann cells, vacuolar myelin degeneration, and even compression of axons also visible. Mild reparative changes and new myelination occurred in Group 2-week (yellow arrows). Active proliferation of Schwann cells was identified in Group 4-week, although degenerated nerve fibres were still present (yellow arrows). At Group 6-week and Group 8-week, the arrangement of nerve fibres gradually returned to normal (yellow arrow), with a large number of newly formed myelin sheaths observed and occasionally with myelosphere changes in myelinated nerve fibres (red arrows).

In the results of transmission electron microscopy, the myelin of the sciatic nerve in the Control was a lamellar structure arranged closely in several layers (Figure 4). The Schwann cells could be seen on one side of the myelin sheath, and their nuclei were large with relatively little cytoplasm. Moreover, an intact axonal membrane was observed, containing a large number of microtubules and microfilaments within the axoplasm. Both myelinated and unmyelinated nerve fibres showed signs of neurodegeneration in the Group 0-week, which was similar to the HE staining. Specifically, there was loose separation of the lamellar structure at the sciatic nerve in the distraction area, with separation of the axonal membrane from the innermost layer of the myelin. The lamellar structure was patchy and loose, exhibiting scattered vacuolated myelin degeneration, compressing and swelling of axons, and even disintegrated myelin fragments. Then, new nerve myelination, active Schwann cell proliferation, and mitochondrial proliferation in the axoplasm were observed in Group 2-week, Group 4-week, and Group 6-week, suggesting that the regenerative phase had started. Finally, in Group 8-week, the injured nerve almost completely recovered to its normal state, and the new myelin was still visible.

**Figure 5:**
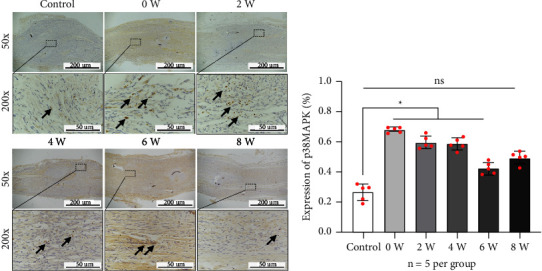
Immunohistochemistry images of p38MAPK in different groups (*n* = 5 per group). (a, b) The semiquantitative analysis indicated that p38MAPK was significantly expressed in Group 2-week, Group 4-week, and Group 6-week compared to the Control (black arrows, *P* < 0.05). However, the expression of p38MAPK was downregulated in the Group 8-week, and there was no statistically significant difference compared to the control group.

In the immunohistochemical analysis (Figure 5(a)), the expression of p38MAPK was significantly increased in Group 0-week (67.84 ± 3.62%), Group 2-week (59.27 ± 1.96%), Group 4-week (51.69 ± 2.54%), and Group 6-week (43.85 ± 2.38%), when compared to the Control (26.71 ± 2.43%, *P* < 0.05). However, there was no statistical difference in the expression of p38MAPK between Group 8-week (29.42 ± 2.43%) and Control (Figure 5(b), *P* > 0.05).

## 4. Discussion

Peripheral nerves are very sensitive to tension stress. Previous studies have reported that the peripheral nerve around the distraction area is usually damaged by the mechanical traction force during the distraction phase, thus hindering the further application of DO [6, 7, 22]. The DO model of the mandible has been well-established and extensively utilized to investigate the mechanism of bone regeneration and remodelling. Studies on mandibular DO models in rabbits have found that denervation is not conducive to bone regeneration in the distraction area. For instance, Zhao et al. [8] successfully established a rabbit mandibular DO model and found that distraction rates of 1.0 and 1.5 mm/d had regenerative effects on the inferior alveolar nerve. Cao et al. [6] observed that loss of the sensory nerves could result in decreased new bone quality during mandibular DO, utilizing their rabbit mandibular DO model. Although these studies have reported changes in peripheral nerves due to different distraction rates during mandibular DO, more attention has focused on the effects of peripheral nerve dysfunction on bone regeneration, rather than the gliding mechanism and repair process of peripheral nerves during DO. Hence, the mechanism of injury and self-repair of peripheral nerves during DO is still unclear, which impedes exploring the development of neural self-repair and regenerative medicine. This study aims to investigate the changes in peripheral nerve self-repair via a rat DO model.

DO technique has been validated for more than three decades, and a DO rate of 1 mm/day is generally accepted in clinical practice for humans, while 0.5 mm/day is widely used for rodent experimental animals, such as SD rats weighing 300 to 400 grams. Ilizarov [23] showed various diameters of nerve axons and irregular cytoplasm in dog tibial peripheral nerve with a lengthening rate of 1 mm/day. However, fewer injurious changes of the nerve were observed when using a DO rate of 0.25 mm/6 h, and the nerve structure was even maintained normal with a total DO rate of 1 mm/day for 60 small turns of the fixator [24, 25]. Namgung et al. [26] reported that nerve rupture occurred when the rabbit sciatic nerve was lengthened by an amplitude of more than 30%–55% of its original length. In this study, a DO rate of 0.5 mm/day was applied in all models, and it was noticed that significant injurious changes in the sciatic nerve were observed near the distraction area via ultrastructure observation. The nerve fibres were disorganized and the nodes of Ranvier were widened after the distraction phase. Lipodrop-like structures produced by the disintegration of Schwann cells were found between nerve fibres, and the myelin sheaths were swollen, which could lead to demyelination of the myelin fibres. The nerve fibres returned to their normal state and the myelin oedema disappeared over time. Therefore, the injurious changes occur during the process of distraction, when the glide of the sciatic nerve is close to the limit of its existing elasticity mechanism. However, the continuity of axons and lamellar structure of the myelin is repaired after 8 weeks of consolidation. We believe that the paraneurium around the sciatic nerve allows it to glide during the distraction phase to reduce the development of injurious changes, which is also the reason why the sciatic nerve can self-repair after the distraction phase, instead of sustaining irreversible damage.

Schwann cells are glial cells of peripheral nerves, which wrap around axons to form myelin in the peripheral nervous system and participate in nerve regeneration [27]. Schwann cells respond rapidly and exhibit plasticity when exposed to peripheral nerve injury, guiding the growth of regenerated axons and promoting peripheral nerve repair and regeneration. It has been reported that the ERK signalling pathway plays an important role in Schwann cell proliferation and migration in the repair process of peripheral nerve, activating the p38 MAPK to regulate Schwann cell elongation and alignment around axons for myelination [28, 29]. It can also upregulate the expression of the transcription factor c-Jun to promote Schwann cells' plasticity and demyelination, transforming them into a repair-promoting phenotype [21, 30]. In this study, the expression of p38MAPK was higher in Group 2-week, Group 4-week, and Group 6-week, compared with the Control (*P* < 0.05), indicating that self-repair of the sciatic nerve had begun after the distraction phase.

The self-repair process of the peripheral nerve gradually progresses during the consolidation phase. However, there is controversy regarding the repair duration of the peripheral nerve after an injury caused by DO. Hirofuji et al. [31] reported that sciatic injurious changes caused by femoral lengthening could be repaired by themselves within 8–12 weeks. Furthermore, the rabbit tibial DO model established by Simpson et al. [7] found that peripheral nerve dysfunction caused by the distraction phase could be recovered approximately 12 weeks after the distraction phase. In this study, the injured nerve recovered after 8 weeks of consolidation. There was no statistically significant difference in MNCV and SFI of the sciatic nerve between the Group 8-week and the Control (*P* > 0.05). We believe that the self-repair duration of the sciatic nerve is related to the distraction rate. Nerves cannot achieve self-repair when the rate and magnitude of distraction exceed the limits of nerve sliding (DO rate > 1.5 mm/day), which can impede bone regeneration and remodelling. Although this study did not compare the repair process after nerve injury caused by different DO rates, based on the observations of this study, we conclude that a distraction rate of 0.5 mm/day is a safe DO rate that contributes to bone regeneration for the rat femoral DO model.

Limitations exist in this study. The physical mechanism of the mechanical force stimulating Schwann cell proliferation and differentiation is still unclear. A vitro experiment is not performed to verify the effects of mechanical forces on Schwann cells. Randomized controlled trials are further needed to elucidate the molecular mechanism between the distraction rate with different amplitudes and nerve elongation in rat peripheral nerves during DO.

## 5. Conclusion

Femoral DO in rats with a rate of 0.5 mm/day may cause sciatic neurapraxia, which can be self-repaired after 8 weeks of consolidation. The paraneurium around the sciatic nerve enables it to glide during the distraction phase to reduce the occurrence of injurious changes. In addition, the plasticity of Schwann cells has been enhanced, leading to a repair-promoting phenotype and forming a microenvironment that is beneficial for sciatic nerve repair and regeneration.

## Data Availability

The original data presented in the study were included in the article/Supplementary Material; further inquiries can be directed to the corresponding author.

## Abbreviations

DO: Distraction osteogenesis
E: Experimental
HE: Hematoxylin-eosin staining
IT: Intermediate toe distance
MNCV: Motor nerve conduction velocity
N: Normal
PL: Footprint length
SD: Sprague –Dawley
SFI: Sciatic function index
SPF: Specific-pathogen-free
TW: Toe width
UK: United Kingdom
US: United States.
